# Clinical outcomes of medical therapy, EUS-guided ethanol ablation, and surgical resection in insulinoma: a clinical epidemiology study

**DOI:** 10.3389/fendo.2026.1848169

**Published:** 2026-07-27

**Authors:** Jin-Ying Lu, Fan Chou, Tsu-Yao Cheng, Fang-Yu Wen, Yu-Wen Tien, Ting-Chun Kuo, Chin-Chen Chang, Wan-Chen Wu, Wei-Yi Chiu, Shyang-Rong Shih, Pei-Lung Chen, Chih-Yuan Wang, Hsiu-Po Wang, Fu-Chang Hu

**Affiliations:** 1 Division of Endocrinology and Metabolism, Department of Internal Medicine, National Taiwan University Hospital, Taipei, Taiwan; 2 Department of Laboratory Medicine, National Taiwan University Hospital, Taipei, Taiwan; 3 Department of Endocrinology and Metabolism, National Taiwan University Hospital, Hsin-Chu Branch, Hsin-Chu, Taiwan; 4 Division of Gastroenterology, Department of Internal Medicine, National Taiwan University Hospital, Taipei, Taiwan; 5 Statistical Consulting Clinic, International-Harvard (I-H) Statistical Consulting Company, Taipei, Taiwan; 6 Division of General Surgery, Department of Surgery, National Taiwan University Hospital, Taipei, Taiwan; 7 Department of Medical Imaging, National Taiwan University Hospital, Taipei, Taiwan; 8 Graduate Institute of Clinical Medicine and School of Nursing, National Taiwan University College of Medicine, Taipei, Taiwan

**Keywords:** ethanol ablation, Firth’s bias-reduced logistic regression model, insulin autoimmune syndrome, insulinoma, somatostatin receptor ligands, surgery

## Abstract

**Introduction:**

Insulinoma is a rare pancreatic neuroendocrine tumor causing hypoglycemia. Surgery is the standard treatment, while endoscopic ultrasound (EUS)-guided ethanol ablation and medical therapy are alternatives in some patients.

**Methods:**

In this retrospective study, we recruited 19 patients with insulinoma treated between 2011 and 2025 at National Taiwan University Hospital. The final treatment strategies were surgery (n = 13), EUS-guided ethanol ablation (n = 3), and medical therapy (n = 3). The study outcomes included post-procedure stays (PPS), treatment-related complications, and follow-up results. New-onset diabetes and complete remission rates for insulinoma were examined and compared across treatment modalities. A Firth’s bias-reduced logistic regression analysis was performed to identify predictors of complete remission in 19 patients with insulinoma.

**Results:**

Patients could receive more than one treatment. Among the three final treatment modalities, surgical resection achieved the highest complete remission rate (13/13; 100%) but was associated with a longer PPS (mean ± standard deviation [SD] = 13.46 ± 7.59 days) and a higher incidence of new-onset diabetes (2/13, 15.38%). EUS-guided ethanol ablation demonstrated moderate efficacy (2/3; 66.67%) with a comparable PPS (18.33 ± 27.43 days). Lastly, medical therapy relieved symptoms but yielded no complete remissions in insulinoma (0/3; 0%). Paradoxical hypoglycemia occurred in 2 of 8 patients (25.0%) during octreotide testing or after direct octreotide treatment. One patient with insulin autoimmune syndrome improved after drug withdrawal and multimodal therapy. Logistic regression analysis identified a positive linear effect of baseline glucose levels (mg/dL) on insulinoma complete remission (estimated adjusted odds ratio [aOR] = 1.2140, *p* = 0.0461) and ever surgery (yes vs. no) as the most effective treatment for complete remission in insulinoma (aOR = 296.0707, *p* = 0.0015).

**Conclusion:**

Although surgery was the most effective treatment, it was associated with longer recovery times and potential metabolic risks. EUS-guided ethanol ablation provided a less invasive alternative with a moderate success rate, whereas medical therapy primarily offered symptomatic control rather than a definitive complete remission. Notably, lower baseline glucose levels were associated with treatment resistance, suggesting a potential role in guiding individualized therapeutic strategies.

## Introduction

Insulinoma is the most common pancreatic neuroendocrine tumor (PNET), characterized by hypoglycemic symptoms resulting from autonomous, inappropriate insulin secretion by pancreatic β-cells (1, 2). Surgical enucleation or partial pancreatectomy is the standard treatment and frequently curative (1). However, surgery carries considerable risks of morbidity and mortality, especially among elderly patients or those with significant comorbidities (3). Medical therapies, including (1) somatostatin receptor ligands (SRLs) (e.g., octreotide or lanreotide) (4, 5) and (2) diazoxide (6), (3) everolimus (7) are often used in patients unfit for surgery, but their efficacy is inconsistent and can be limited by adverse effects (8, 9).

Endoscopic ultrasound (EUS)-guided ethanol ablation has emerged as a novel and minimally invasive approach to managing small functional PNETs, particularly insulinomas (10). This technique offers the potential to alleviate symptoms and control tumors with less procedural risk. However, evidence on its long-term safety and efficacy, particularly compared to surgery, remains limited (11). Moreover, predictors of insulinoma complete remission have not been well characterized in previous studies.

In this retrospective study, we recruited 19 patients diagnosed with insulinoma, including 5 who had undergone EUS-guided ethanol ablation. The clinical outcomes were evaluated and compared with those receiving surgical or medical therapies. A literature review was conducted to assess the currently available evidence on EUS-guided ethanol ablation for insulinomas to contextualize our findings. Most importantly, given three treatment strategies, this clinical epidemiological study aimed to identify predictors of complete remission for insulinoma.

## Methods

### Study design and patient selection

**Figure 1:**
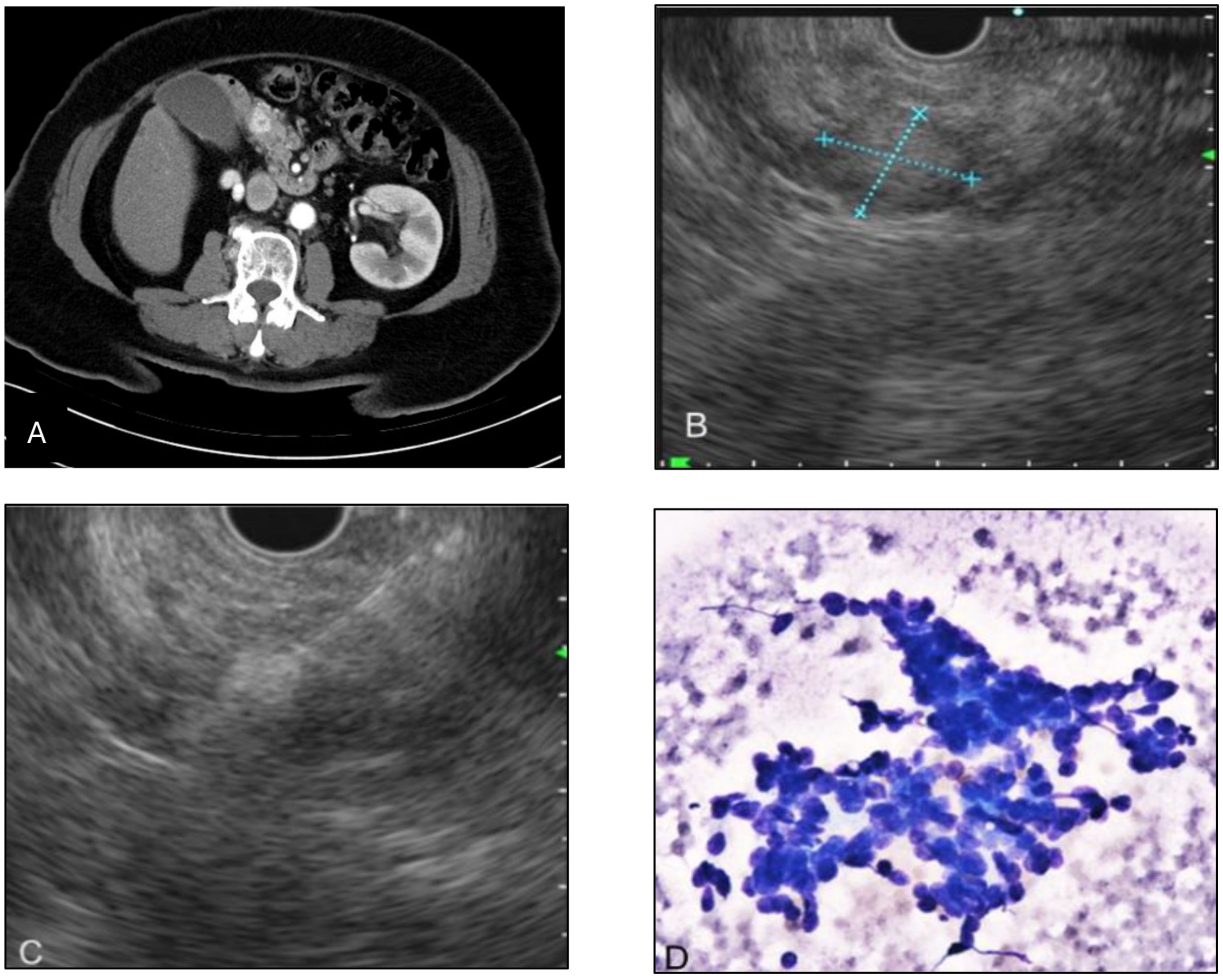
Illustrative graphs. **(A)** Axial abdominal post-contrast computed tomography showed at least one hypervascular nodule measuring up to 1.5 cm in the pancreas. **(B)** Endoscopic ultrasonography (EUS) revealed a hypoechoic, homogeneous lesion in the pancreatic head. **(C)** EUS-guided fine-needle injection using a 22-gauge needle with 99% ethanol was performed until a hyperechoic blush was observed within the tumor. **(D)** Histopathology showed small, round, blue cells consistent with a pancreatic neuroendocrine tumor.

This study was approved by our Institutional Review Board (Approval Number: 202504107RINC). As listed in **Table 1**, the series of 19 patients with insulinoma was retrospectively collected at the National Taiwan University Hospital from December 2011 to April 2025. Patients with suspected insulinoma were identified based on clinical presentation and imaging findings. The inclusion criteria were: (1) signs and symptoms consistent with hypoglycemia, (2) plasma glucose ≤ 45 mg/dL accompanied by insulin > 3 µIU/mL, and (3) prompt resolution of symptoms following glucose administration. The imaging criteria for insulinoma consisted of well-defined hypervascular pancreatic lesions on contrast-enhanced computed tomography (CT) or magnetic resonance imaging (MRI). In cases with inconclusive CT/MRI results, EUS or selective arterial calcium stimulation test (SACST) with hepatic venous sampling was used to localize the lesion. An octreotide challenge test (OCT) was performed to assess eligibility for somatostatin receptor ligands (SRLs) therapy. The diagnostic and therapeutic workflow for a representative case of insulinoma is illustrated in **Figure 1**, including contrast-enhanced CT findings, EUS-based identification of the lesion, ethanol injection under EUS guidance, and histopathological confirmation of a PNET.

**Table 1:** Demographic and clinical characteristics, specialized examinations, treatment modalities, and follow-up outcomes in 19 patients with insulinoma.

Patient	Gender	Age^1^	SACS	OCT	Tumor size (mm)	Tumor location	Number	Treatment	Complication^3^
1	Female	73	Negative	Euglycemia	15	Head, neck	6	Ethanol ablation; monthly octreotide LAR	None
2	Female	57	Positive GDA	Euglycemia	12	Head	1	Ethanol ablation; Pancreatic tumor enucleation	Fistula
3	Female	68	Positive SMA	Euglycemia	9	Head	1	Ethanol ablation	None
4	Female	44			12−17	Head, body	2	Ethanol ablation	None
5	Female	51			19	Neck	1	Distal pancreatectomy	None
6	Female	35	Positive SMA/GDA		9	Neck	1	Pancreatic tumor enucleation	None
7	Female	47	Positive SMA	Euglycemia	5−10	Head^2^	2	Pancreatectomy	P-J leakage
8	Female	59			12	Body	1	Pancreatico-duodenectomy	None
9	Female	86			6−20	Body, neck	2	Pancreatectomy	None
10	Male	58			46	Head	1	Pancreatectomy	None
11	Female	50			11	Head	1	Pancreatic enucleation	P-J leakage
12	Male	29			18	Head	1	Pancreatic enucleation	None
13	Male	87			9−13	Head, body	2	Pancreatic enucleation	Infection
14	Female	53			13	Head	1	Pancreatic enucleation	None
15	Female	59			18	Uncinate process	1	Pancreatic enucleation	P-J leakage
16	Male	80		Euglycemia (after directly receiving octreotide therapy)	9	Body	1	Discontinuation of clopidogrel; ethanol; octreotide LAR	None
17	Male	58		Paradoxical hypoglycemia	52	Tail plus multiple liver metastases	Numerus	Lanreotide plus everolimus	None
18	Female	58		Euglycemia	22	Body	1	Octreotide LAR	None
19	Male	59		Paradoxical hypoglycemia	27	Tail	1	Distal pancreatectomy	None

OCT, octreotide test; SACS, selective arterial calcium stimulation and hepatic venous sampling; GDA, gastroduodenal artery; SMA, superior mesenteric artery; LAR, long-acting release; P-J, pancreaticojejunostomy.

^1^ Age of being diagnosed with insulinoma.

^2^ Near the pancreatic duct.

^3^ P-J leakage refers to pancreaticojejunostomy leakage, a complication that can occur after pancreatic surgery. Both infection and fistula formation are recognized as post-surgical complications.

### Pathological and/or clinical confirmation

Histological confirmation was obtained by EUS-guided fine-needle aspiration (FNA), core needle biopsy (CNB), or surgical excision, according to the WHO 2020 classification (12). Before treatment selection, all patients underwent diagnostic evaluation to confirm the presence of endogenous hyperinsulinemic hypoglycemia and to localize the suspected insulinoma (13). Careful preoperative differential diagnosis is essential to avoid unnecessary treatment of other pancreatic lesions that may mimic insulinoma (14). Patients were excluded if they had a known allergy to ethanol, if the tumor could not be visualized or accessed under EUS guidance, or if they had active acute pancreatitis. A patient, who had a recent myocardial infarction and was on dual-antiplatelet therapy, was considered unsuitable for biopsy and was diagnosed based on clinical presentation, laboratory results, and imaging findings.

### Interventions

Patients could receive more than one treatment due to treatment failure. The surgical treatment consisted of pancreatic enucleation, distal pancreatectomy, or Whipple procedure (pancreaticoduodenectomy). Medical therapy included diazoxide, somatostatin receptor ligands (SRLs) including octreotide and lanreotide, or everolimus. Among the 19 patients with insulinoma, the first treatments chosen were (1) surgery (*n* = 14; 73.68%), (2) ethanol ablation ± octreotide (*n* = 3; 15.79%), and (3) medical therapy (*n* = 2; 10.53%), the *final* treatments were (1) surgery (*n* = 13; 68.42%), (2) ethanol ablation ± octreotide (*n* = 3; 15.79%), and (3) medical therapy (*n* = 3; 15.79%), and the treatments ever received were (1) surgery (*n* = 15/19; 78.95%), (2) ethanol ablation ± octreotide (*n* = 5/19; 26.32%), and (3) medical therapy (*n* = 3/19; 15.79%), respectively (**Table 2**). Specifically, five patients who were not suitable or unwilling to undergo surgery or who experienced recurrent hypoglycemia after surgery had ever received EUS-guided ethanol ablation. Two patients received medical therapy alone, and one other patient received adjunctive medical therapy following ethanol ablation.

**Table 2:** Comparison of patient characteristics and outcomes among the final treatment modalities.^1^^, ^^2^

Variable^3^	All patients	1. Surgery (Enucleation, distal pancreatectomy, Whipple)^1^	2. Ethanol ablation ± octreotide^1^	3. Medical therapy (Octreotide or lanreotide alone)^1^	P value^2^
Number of subjects (n)^3^	19 (100%)	13 (68.42%)	3 (15.79%)	3 (15.79%)	
Age (years)	58.68 ± 15.20 [51.35–66.01]	56.15 ± 16.36 [46.26–66.04]	65.33 ± 16.17 [25.16–105.50]	63.00 ± 8.66 [41.49–84.51]	0.5725
Gender					>0.9999
Male	6 (31.58%)	4 (30.77%)	1 (33.33%)	1 (33.33%)	
Female	13 (68.42%)	9 (69.23%)	2 (66.67%)	2 (66.67%)	
SACST					>0.9999
No	15 (78.95%)	10 (76.92%)	2 (66.67%)	3 (100%)	
Yes	4 (21.05%)	3 (23.08%)	1 (33.33%)	0 (0%)	
OCT					0.0720
No	12 (63.16%)	10 (76.92%)	2 (66.67%)	0 (0%)	
Yes	7 (36.84%)	3 (23.08%)	1 (33.33%)	3 (100%)	
Tumor size (cm)	1.89 ± 1.20 [1.31–2.47]	1.85 ± 1.01 [1.24–2.46]	1.00 ± 0.17 [0.58–1.42]	2.97 ± 1.97 [−1.92–7.86]	0.0506
Fasting plasma glucose (mg/dL) at baseline	38.58 ± 8.96 [34.26–42.90]	38.23 ± 8.48 [33.11–43.35]	44.67 ± 4.62 [33.19–56.15]	34.00 ± 13.53 [0.39–67.61]	0.3036
HbA1c (%) at baseline	5.01 ± 0.49 [4.77–5.25]	4.83 ± 0.46 [4.55–5.11]	5.70 ± 0.42 [4.66–6.74]	5.07 ± 0.06 [4.92–5.22]	0.0729
Number of missing subjects (*n*)^3^		4	1	0	
Insulin (μIU/mL) at baseline	73.54 ± 133.71 [9.09–137.99]	39.42 ± 35.73 [17.83–61.01]	211.50 ± 336.48 [−624.36–1047.36]	83.43 ± 61.30 [−68.85–235.71]	0.4280
C-peptide level (ng/mL) at baseline	5.34 ± 4.31 [3.26–7.42]	4.44 ± 2.35 [3.02–5.86]	3.19 ± 0.83 [1.13–5.25]	11.39 ± 8.15 [−8.86–31.64]	0.1470
Fasting plasma glucose (mg/dL) after treatment	97.89 ± 13.02 [91.61–104.17]	99.33 ± 10.71 [92.86–105.80]	101.67 ± 24.58 [40.61–162.73]	88.33 ± 5.86 [73.77–102.89]	0.3337
Number of missing subjects (*n*)^3^		1	0	0	
HbA1c (%) after treatment	5.96 ± 0.57 [5.69–6.23]	6.00 ± 0.60 [5.64–6.36]	6.13 ± 0.49 [4.91–7.35]	5.63 ± 0.59 [4.17–7.09]	0.6087
Number of missing subjects (*n*)^3^		2	0	0	
Insulin (μIU/mL) after treatment	7.75 ± 4.43 [5.61–9.89]	7.65 ± 4.38 [5.00–10.30]	8.74 ± 7.11 [−8.92–26.40]	6.97 ± 2.62 [0.46–13.48]	0.9952
Number of missing subjects (*n*)^3^		2	0	0	
C-peptide level (ng/mL) after treatment	1.78 ± 0.63 [1.48–2.08]	1.82 ± 0.63 [1.44–2.20]	2.05 ± 0.79 [0.09–4.01]	1.45 ± 0.55 [0.08–2.82]	0.4789
Number of missing subjects (*n*)^3^		9	0	0	
First treatment					0.0106
Surgery	14 (73.68%)	12 (92.31%)	1 (33.33%)	1 (33.33%)	
Ethanol ablation ± Octreotide	3 (15.79%)	1 (7.69%)	2 (66.67%)	0 (0%)	
Medical therapy	2 (10.53%)	0 (0%)	0 (0%)	2 (66.67%)	
Ever surgery					0.0039
No	4 (21.05%)	0 (0%)	2 (66.67%)	2 (66.67%)	
Yes	15 (78.95%)	13 (100%)	1 (33.33%)	1 (33.33%)	
Ever ethanol ablation ± Octreotide					0.0072
No	14 (73.68%)	12 (92.31%)	0 (0%)	2 (66.67%)	
Yes	5 (26.32%)	1 (7.69%)	3 (100%)	1 (33.33%)	
Ever medical therapy					0.0021
No	16 (84.21%)	13 (100%)	2 (66.67%)	0 (0%)	
Yes	3 (15.79%)	0 (0%)	1 (33.33%)	3 (100%)	
Hospital stays (days)	21.95 ± 12.13 [16.10–27.80]	22.15 ± 7.72 [17.48–26.82]	31.00 ± 26.29 [−34.31–96.31]	12.00 ± 1.73 [7.70–16.30]	0.0468
Post-procedure stays (PPS) (days)	13.61 ± 11.74 [7.95–19.27]	13.46 ± 7.59 [8.87–18.05]	18.33 ± 27.43 [−49.80–86.46]	7.50 ± 2.12 [2.23–12.77]	0.2399
Number of missing subjects (*n*)^3^				1	
Complete remission rate^4^	15/19 (78.95%)	13/13 (100%)	2/3 (66.67%)	0/3 (0%)	0.0015
Follow-up (months)	77.18 ± 51.08 [52.56–101.80]	77.39 ± 57.14 [42.86–111.92]	96.64 ± 27.65 [27.95–165.33]	56.84 ± 44.05 [−52.59–166.27]	0.8048
Newly onset diabetes mellitus^5^	3/19 (15.79%)	2/13 (15.38%)	1/3 (33.33%)	0/3 (0%)	0.7049

^1^ The three final treatment groups were classified according to the final therapeutic modality that achieved euglycemia or near-euglycemia during follow-up.

^2^ Continuous variables are presented as mean ± standard deviation (SD) with 95% confidence intervals (95% CIs) in brackets, and categorical variables are presented as frequency (percentage, %). P-values were calculated using the Kruskal–Wallis rank-sum test for continuous variables and Fisher’s exact test for categorical variables because of the small sample size.

^3^ Missing values were present for six variables. The number of missing subjects (n) is indicated in the row immediately below the corresponding variable.

^4^ Complete remission was defined as sustained euglycemia without the need for anti-hypoglycemic medication or additional therapeutic intervention for at least 1 year after treatment. The numerator indicates the number of *complete remission* patients, and the denominator indicates the number of patients treated with the respective modality.

^5^ New-onset diabetes mellitus was defined as an HbA1c level ≥6.5% at any time during follow-up.

HbA1c, Glycated hemoglobin; OCT, Octreotide test; SACST, Selective arterial calcium stimulation test.

EUS-guided ethanol ablation was performed using a standardized approach based on previously described techniques (15). Under real-time visualization with a linear array echoendoscope, the tumor was targeted and punctured using a 22- or 25-gauge needle. Ablation was achieved via fine-needle injection of 99% ethanol. The injection was delivered slowly while gradually withdrawing the needle from the deepest portion of the lesion, halting once a hyperechoic blush extended to the tumor border. To ensure complete ablation under continuous EUS evaluation, repeat injections were performed, when necessary, carefully avoiding the previous puncture tract. The total volume of ethanol injected was dynamically determined by real-time ultrasound changes observed within the tumor. Treatment efficacy was monitored through follow-up EUS or radiological imaging alongside clinical symptom assessment. Treatment success was defined as complete radiological ablation with complete resolution of hypoglycemic symptoms.

All 19 patients were categorized according to their final therapeutic modalities that achieved euglycemia or near-euglycemia during follow-up. Procedural outcomes, adverse events, and treatment efficacy were compared among surgery, ethanol ablation, and medical therapy. The complete remission in insulinoma was defined as sustained biochemical euglycemia (fasting plasma glucose ≥70 mg/dL) together with complete resolution of hypoglycemic symptoms for at least 1 year after treatment, without the need for anti-hypoglycemic medications or additional therapeutic intervention.

### Statistical analysis

All analyses were performed using the R software, version 4.4.1 (R Foundation for Statistical Computing, Vienna, Austria). A two-sided *p* value ≤ 0.05 was considered statistically significant. Continuous variables are presented as mean ± SD with confidence intervals in brackets and categorical variables as frequencies and percentages (%). Power transformations (*x^q^*), including the natural logarithm (*q* = 0), square root (*q* = 0.5), and squared (*q* = 2), were applied to some continuous variables to make their distributions more symmetric.

Univariate analyses of pre- and post-treatment variables among the three final treatment modalities were conducted using the Wilcoxon rank-sum test, Chi-squared test, or Fisher’s exact test, as appropriate for the data type. Distributions of glucose, glycated hemoglobin (HbA1c), insulin, and C-peptide levels before and after treatment were visualized and compared by drawing the box plots across the three final treatment modalities and the final treatment outcome (complete remission vs. non-complete remission). Paired comparisons were performed using a one-tailed Wilcoxon signed-rank test, as directional changes were prespecified: increases in glucose and HbA1c, and decreases in insulin and C-peptide, were expected following treatment. Multivariate analysis was performed by fitting logistic regression models to estimate the adjusted effects of treatment modality and relevant baseline covariates on the probability of complete remission from insulinoma. In addition, all treatment modalities received by each patient (first, ever, and final treatment modalities) were considered in the regression analysis to account for treatment heterogeneity. To ensure a good quality of the analysis result, the model-fitting techniques for (1) variable selection, (2) goodness-of-fit (GOF) assessment, and (3) regression diagnostics and remedies were applied in our logistic regression analysis, which was described in the **Supplementary Appendix**.

## Results

We listed the demographic and clinical characteristics, specialized examinations, treatment modalities, and follow-up outcomes for all 19 patients with insulinoma in **Table 1**. Next, we compared patient characteristics and treatment outcomes across the three final treatment modalities that achieved euglycemia or near-euglycemia during the follow-up period in **Table 2**, and between the complete remission and non-complete remission groups in **Table 3**. For example, if a patient did not achieve remission with surgery but experienced resolution of hypoglycemia following ethanol injection, this patient was classified in the final ethanol ablation group. Finally, we presented the results of a Firth’s bias-reduced logistic regression analysis of insulinoma complete remission to identify its predictors for guidance in clinical practice in **Table 4**.

**Table 3:** Comparison of patient characteristics and outcomes between the complete remission and non-complete remission groups.^1^^, ^^2^.

Variable^3^	All patients	Non-complete remission	Complete remission^1^	P value^2^
Number of subjects (n)^3^	19 (100%)	4 (21.05%)	15 (78.95%)	
Age of diagnosis (years)	58.68 ± 15.20 [51.35–66.01]	67.25 ± 11.06 [49.67–84.83]	56.40 ± 15.63 [47.74–65.06]	0.2097
Gender				0.5573
Male	6 (31.58%)	2 (33.33%)	4 (66.67%)	
Female	13 (68.42%)	2 (15.38%)	11 (84.62%)	
SACST				0.5304
No	15 (78.95%)	4 (26.67%)	11 (73.33%)	
Yes	4 (21.05%)	0 (0%)	4 (100%)	
OCT				0.1174
No	12 (63.16%)	1 (8.33%)	11 (91.67%)	
Yes	7 (36.84%)	3 (42.86%)	4 (57.14%)	
Tumor size (cm)	1.89 ± 1.20 [1.31–2.47]	2.45 ± 1.91 [−0.59–5.49]	1.75 ± 0.98 [1.21–2.29]	0.5810
Fasting plasma glucose (mg/dL) at baseline	38.58 ± 8.96 [34.26–42.90]	36.00 ± 11.75 [17.27–54.73]	39.27 ± 8.45 [34.59–43.95]	0.6158
HbA1c (%) at baseline	5.01 ± 0.49 [4.77–5.25]	5.30 ± 0.47 [4.55–6.05]	4.89 ± 0.47 [4.63–5.15]	0.1555
Number of missing subjects (*n*)^3^		0	5	
Insulin (μIU/mL) at baseline	73.54 ± 133.71 [9.09–137.99]	212.57 ± 263.09 [−206.39–631.53]	36.46 ± 34.03 [17.62–55.30]	0.0624
C-peptide level (ng/mL) at baseline	5.34 ± 4.31 [3.26–7.42]	9.25 ± 7.92 [−3.33–21.83]	4.30 ± 2.23 [3.06–5.54]	0.3070
Fasting plasma glucose (mg/dL) after treatment	97.89 ± 13.02 [91.61–104.17]	97.50 ± 18.95 [67.36–127.64]	98.00 ± 11.78 [91.48–104.52]	0.7100
Number of missing subjects (*n*)^3^		0	1	
HbA1c (%) after treatment	5.96 ± 0.57 [5.69–6.23]	5.90 ± 0.72 [4.75–7.05]	5.98 ± 0.55 [5.67–6.29]	>0.9999
Number of missing subjects (*n*)^3^		0	2	
Insulin (μIU/mL) after treatment	7.75 ± 4.43 [5.61–9.89]	6.22 ± 2.60 [2.08–10.36]	8.52 ± 5.09 [5.70–11.34]	0.4962
Number of missing subjects (*n*)^3^		0	7	
C-peptide level (ng/mL) after treatment	1.78 ± 0.63 [1.48–2.08]	1.57 ± 0.51 [0.76–2.38]	1.92 ± 0.71 [1.53–2.31]	0.6095
Number of missing subjects (*n*)^3^		0	9	
First treatment modalities				0.0157
Surgery	14 (73.68%)	1 (7.14%)	13 (92.86%)	
Ethanol ablation ± Octreotide	3 (15.79%)	1 (33.33%)	2 (66.67%)	
Medical therapy	2 (10.53%)	2 (100%)	0 (0%)	
Ever surgery				0.0157
No	4 (21.05%)	3 (75.00%)	1 (25.00%)	
Yes	15 (78.95%)	1 (6.67%)	14 (93.33%)	
Ever ethanol ablation ± Octreotide				0.2722
No	14 (73.68%)	2 (14.29%)	12 (85.71%)	
Yes	5 (26.32%)	2 (40.00%)	3 (60.00%)	
Ever medical therapy				0.0041
No	16 (84.21%)	1 (6.25%)	15 (93.75%)	
Yes	3 (15.79%)	3 (100%)	0 (0%)	
Hospital stays (days)	21.95 ± 12.13 [16.10–27.80]	24.25 ± 24.54 [−14.77–63.27]	21.33 ± 7.62 [17.11–25.55]	0.2087
Post-procedure stays (PPS) (days)	13.61 ± 11.74 [7.95–19.27]	21.67 ± 24.58 [−17.48–60.82]	12.00 ± 8.02 [7.55–16.45]	>0.9999
Number of missing subjects (*n*)^3^		1	0	
Follow-up (months)	77.18 ± 51.08 [52.46–101.90]	63.52 ± 38.37 [2.41–124.63]	80.83 ± 54.52 [50.67–110.99]	0.7262
Newly onset diabetes mellitus (DM)^4^	3/19 (15.79%)	1/4 (25.00%)	2/15 (13.33%)	0.5304
Final treatment modalities				0.0015
Surgery	13 (68.42%)	0 (0%)	13 (100%)	
Ethanol ablation	3 (15.79%)	1 (33.33%)	2 (66.67%)	
Medicine	3 (15.79%)	3 (100%)	0 (0%)	

^1^ Complete remission was defined as remaining free from anti-hypoglycemic medications and additional therapeutic interventions for at least 1 year following treatment.

^2^ Continuous variables are presented as mean ± standard deviation (SD) with 95% confidence intervals (95% CIs) in brackets, and categorical variables are presented as frequency (percentage, %). P-values were calculated using the Wilcoxon rank-sum test for continuous variables and Fisher’s exact test for categorical variables due to the small sample size.

^3^ Six variables had missing values in some subjects. The number of missing subjects (n) is shown in the row immediately below the corresponding variable.

^4^ New-onset diabetes mellitus was defined as an HbA1c level ≥6.5% at any time during the follow-up period.

HbA1c, Glycated hemoglobin; OCT, Octreotide test; SACST, Selective arterial calcium stimulation test.

**Table 4:** A multivariate analysis by fitting the Firth’s bias-reduced logistic regression model to identify predictors of complete remission in insulinoma.^1^.

Covariate^2^	Estimated regression coefficient	Estimated standard error	*χ* value	*p* value	Estimated odds ratio	95% confidence interval of odds ratio
Intercept	–9.6129	5.1535	4.6121	0.0317	0.0001	0.0000−0.4966
Fasting plasma glucose (mg/dL) at baseline^3^	0.1940	0.1078	3.9763	0.0461	1.2140	1.0029−2.1688
Ever surgery (yes vs. no)	5.6906	2.6068	10.0952	0.0015	296.0707	5.3094−3307488629.6502

^1^ A Firth’s bias-reduced logistic regression model was used because of quasi-separation/separation issues in the covariates “ever surgery” and “ever medical therapy.” Model discrimination was assessed by the area under the receiver operating characteristic curve (AUC = 0.9667; 95% CI, 0.8876–1.000).

^2^ Candidate covariates included treatment modality (first, ever, and final treatment), age, sex, baseline glucose level, insulin, C-peptide level, and tumor size. Variable selection was performed using a stepwise procedure.

^3^ A generalized additive model (GAM) analysis demonstrated a linear association between baseline glucose level and the logit probability of complete remission.

### Demographic characteristics

As shown in **Table 2**, females comprised approximately 66–69% of patients across the three final treatment groups (*p* > 0.9999). Surgical patients were generally younger (56.15 ± 16.36 years), whereas those receiving EUS-guided ethanol ablation (65.33 ± 16.17 years) or medical therapy (63.00 ± 8.66 years) were older, reflecting potential age- or comorbidity-related limitations in surgical eligibility (*p* = 0.5725).

### Special diagnostic procedures

In **Table 2**, we observed that the SACST utilization rate was highest in the final ethanol ablation group (1/3, 33.3%) and lowest in the final medical therapy group (0/3, 0%), while the final surgery group had an intermediate rate (3/13, 23.1%) (*p* > 0.9999). Conversely, the use of the octreotide test (OCT) was most frequent in the final medical therapy group (3/3, 100%) and least common in the final surgical group (3/13, 23.1%), while the final ethanol ablation group had a use rate of 33.3% (1/3) (*p* = 0.0720). SACST successfully localized the pancreatic tumor in 4 of 5 patients (80%) and was useful for the functional evaluation of tumor localization. Notably, 2 out of 8 patients (25.0%) undergoing OCT or following direct octreotide therapy exhibited paradoxical hypoglycemia, despite adequate insulin suppression during the procedure (**Table 1**). These findings suggested that SACST was performed preferentially prior to ethanol ablation, whereas OCT was used more frequently before medical therapy. Specifically, OCT is strongly recommended before initiating medical therapies to identify patients at risk of severe hypoglycemia.

### Tumor size

Tumor size varied in 19 patients (1.89 ± 1.20 cm) and differed almost significantly across the three final treatment groups (*p* = 0.0506), suggesting that tumor size might influence the final treatment choice. The final medical therapy group had the largest tumors (2.97 ± 1.97 cm), likely reflecting inoperability; the final surgery group had moderately sized tumors (1.85 ± 1.01 cm); and the final ethanol ablation group had the smallest (1.00 ± 0.17 cm), favoring the feasibility of a minimally invasive approach (**Tables 1**, **2**).

### Biochemical profiles before treatment

All 19 insulinoma patients presented with symptomatic hypoglycemia. At baseline, they had low fasting plasma glucose (38.58 ± 8.96 mg/dL; range: 20−53 mg/dL), which did not differ significantly across the three final treatment groups (*p* = 0.3036), and low HbA1c levels (5.01 ± 0.49%; range: 4.3–6.0%), of which the lowest was in the final surgical group (*p* = 0.0729). Elevated insulin (73.54 ± 133.71 μIU/mL; range: 3.9– >600 μIU/mL) and C-peptide levels (5.34 ± 4.31 ng/mL; range: 1.46–20.00 ng/mL), of which both did not differ significantly across the three final treatment groups (*p* = 0.4280 and 0.1470), confirmed endogenous hyperinsulinemia consistent with insulinoma.

### Patient outcomes of final treatment modalities

In **Table 2**, we found that the 19 insulinoma patients had a hospital stay of 21.95 ± 12.13 days, post-procedure stay (PPS) of 13.61 ± 11.74 days, an estimated complete remission rate of 78.95% (15/19), and a follow-up duration of 77.18 ± 51.08 months. Only hospital stay (days) and complete remission rate differed significantly across the three final treatment modalities (*p* = 0.0468 and 0.0015).

Patients who finally underwent surgical resection, including tumor enucleation, distal pancreatectomy, or pancreaticoduodenectomy (Whipple procedure), had a long hospital stay of 22.15 ± 7.72 days (median: 21 days) and PPS of 13.46 ± 7.59 days (median: 11 days) for recovery. This final treatment group achieved a 100% complete remission rate (13/13). The follow-up period was 77.39 ± 57.14 months (median: 82.43 months).

Next, patients who were ultimately treated with EUS-guided ethanol ablation, alone or in combination with SRLs, had a prolonged hospital stay of 31.00 ± 26.29 days (median: 20 days) and a prolonged PPS of 18.33 ± 27.43 days (median: 3 days). Hospital stays and recovery times varied a lot, depending on comorbidities and adjunctive treatments. The complete remission rate was 66.67% (2/3). The follow-up period was 96.64 ± 27.65 months (median: 83.58 months).

Finally, patients who received medical therapy alone, such as octreotide or lanreotide, had the shortest hospital stay of 12.00 ± 1.73 days (median: 11 days) and PPS of 7.50 ± 2.12 days (median: 7.50 days) with one missing value. None of these patients (0/3) were in complete remission, but they achieved symptomatic control. The follow-up period was 56.84 ± 44.05 months (median: 59.10 months).

Specifically, the following cases deserved close attention.

- Two patients achieved complete and sustained resolution of hypoglycemia without further interventions, including one who received ethanol ablation as a rescue treatment after the surgery failed.
- A patient initially treated with ethanol ablation experienced recurrent hypoglycemia and subsequently achieved euglycemia following surgical enucleation of a pancreatic head tumor.
- Another patient with multifocal insulinomas achieved complete symptom relief with adjunctive octreotide long-acting release therapy. This individual also experienced a marked weight reduction from 101 kg to 51 kg within 12 months of treatment initiation and maintained this benefit for more than 100 months.
- One patient had coexisting clopidogrel-induced insulin autoimmune syndrome (IAS) and insulinoma and responded well to a combination of withdrawal of medications, ethanol ablation, and octreotide LAR therapy.

### New-onset diabetes mellitus after treatment

New-onset DM following treatment was defined as an HbA1c level exceeding 6.5% during follow-up, which was uncommon (**Table 2**). No such cases were observed among patients who received medical therapy alone, but two patients developed DM after distal pancreatectomy in the surgery group (2/13, 15.38%), and an elderly patient developed DM following clopidogrel discontinuation (due to insulin autoimmune syndrome), ethanol ablation, and SRL therapy in the ethanol ablation group (1/3, 33.33%) (*p* = 0.7049). These findings suggested that old age, extensive pancreatic surgery, or multimodal therapeutic strategies may increase the metabolic risk such as the onset of DM (3, 16).

### Analysis of biochemical changes after treatment

**Figure 2:**
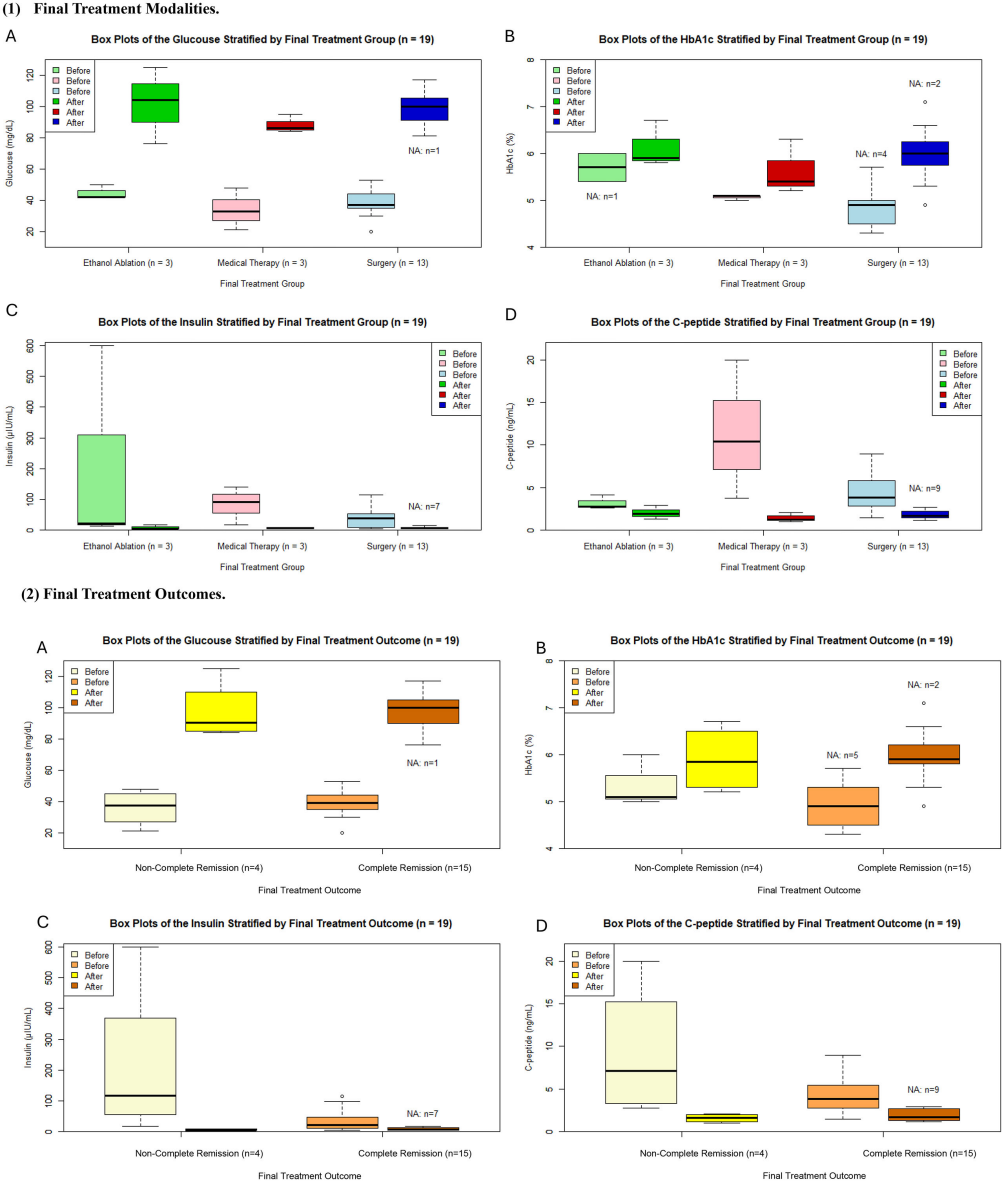
Box plots of glycemic and hormonal parameters stratified by final treatment strategies, and final treatment outcomes (*n* = 19). (1) Final Treatment Strategies: Each panel displays before and after treatment levels of key metabolic markers in three final treatment groups: Ethanol Ablation (*n* = 3), Medical Therapy (*n* = 3), and Surgery (*n* = 13). **(A)** Glucose increased after treatment, observed in all three treatment groups (*p* = 0.0379, 0.0050, and < 0.0001 in *ethanol ablation*, *medical therapy*, and *surgery*, respectively). **(B)** HbA1c levels increased after treatment. When stratified by final treatment group, the increase was statistically significant only in the *surgery* group (*p* = 0.0003). **(C)** Insulin levels decreased after treatment, reflecting suppressed insulin secretion or improved clearance. The reduction was significant only in the *surgery* group (*p* = 0.0323). **(D)** C-peptide levels decreased after treatment, suggesting reduced endogenous insulin secretion. The reduction was significant only in the *ethanol ablation* group (*p* = 0.0075). Box plots show medians, interquartile ranges, and extreme observations. Missing data are indicated as “NA” with corresponding counts. Distinct colors represent “Before” and “After” treatment for each final treatment group, which were examined using a one-tailed Wilcoxon signed-rank test for paired data. (2) Final Treatment Outcomes: Box plots of glycemic and hormonal parameters before and after treatment, stratified by the final treatment outcomes: Non-Complete Remission (*n* = 4), and Complete Remission (*n* = 15). **(A)** Even among patients who were *not in complete remission* (*n* = 4), glucose levels increased after treatment (p = 0.0024). Patients who were *in complete remission* (*n* = 15) likewise showed higher post-treatment glucose levels (p < 0.0001). One post-treatment glucose measurement was missing in the complete remission group. **(B)** HbA1c increased after treatment even in the *non-complete remission* group (*p* = 0.0388) and increased even more markedly in the *complete remission* group (*p* = 0.0003). Two post-treatment HbA1c values were missing in the *complete remission* group. **(C)** Insulin levels did not significantly decrease after treatment in the *non-complete remission* group but decreased significantly in the *complete remission* group (p = 0.0193). Seven post-treatment insulin measurements were missing. **(D)** In both the *non-complete remission* and *complete remission* groups, c-peptide levels decreased with borderline significance (*p* = 0.0789 and 0.0567, respectively). Nine post-treatment c-peptide values were missing in the *complete remission* group. NA: missing values as indicated on each panel. Distinct colors represent “Before” and “After” treatment for each final treatment outcome group, which were examined using a one-tailed Wilcoxon signed-rank test for paired data. A one-tailed approach was prespecified based on the expected direction of change: glucose and HbA1c were anticipated to increase after treatment, whereas insulin and C-peptide were expected to decrease.

In **Table 2** and **Figure 2** (1), post-treatment laboratory findings showed significant reductions in fasting plasma glucose levels across all three final treatment groups. The fasting plasma glucose after treatment was 97.89 ± 13.02 mg/dL in all 19 patients, with 99.33 ± 10.71, 101.67 ± 24.58, and 88.33 ± 5.86 mg/dL in the final surgery, ethanol ablation, and medical therapy groups, respectively (*p* = 0.3337). Although HbA1c levels also increased after treatment, the change was modest, likely due to the long half-life of HbA1c and to previously suppressed baseline levels resulting from chronic intermittent hypoglycemia. The HbA1c (%) after treatment was 5.96 ± 0.57 in all 19 patients, with 6.00 ± 0.60, 6.13 ± 0.49, and 5.63 ± 0.59 in the final surgery, ethanol ablation, and medical therapy groups, respectively (*p* = 0.6087). Insulin and C-peptide concentrations generally decreased after treatment. The wide variation in pre-treatment insulin levels may, in part, be explained by falsely elevated insulin concentrations in patients with IAS, where circulating insulin autoantibodies interfered with the test’s accuracy. The insulin after treatment was 7.75 ± 4.43 μIU/mL in all 19 patients, with 7.65 ± 4.38, 8.74 ± 7.11, and 6.97 ± 2.62 μIU/mL in the final surgery, ethanol ablation, and medical therapy groups, respectively (*p* = 0.9952). And the C-peptide level after treatment was 1.78 ± 0.63 ng/mL in all 19 patients, with 1.82 ± 0.63, 2.05 ± 0.79, and 1.45 ± 0.55 ng/mL in the final surgery, ethanol ablation, and medical therapy groups, respectively (*p* = 0.4789). As noted in **Table 2**, several post-treatment biochemical measurements were missing in the final surgery group.

Moreover, **Figure 2** (1) shows changes and improvements in glycemic and hormonal parameters before and after treatment across the three final treatment groups. Specifically, the changes in fasting plasma glucose after treatment significantly differed from 0 in the final ethanol ablation, medical therapy, and surgery groups (*p* = 0.0379, 0.0050, and < 0.0001, respectively). The increase in HbA1c level after treatment was statistically significant only in the surgery group (*p* = 0.0003). The reduction of insulin levels after treatment, reflecting suppressed insulin secretion or improved clearance, was also statistically significant in the surgery group (*p* = 0.0323). However, the decline in C-peptide level after treatment, suggesting reduced endogenous insulin secretion, was statistically significant only in the ethanol ablation group (*p* = 0.0075). Although notable improvements were observed in the box plots of **Figure 2** (1), some changes did not reach statistical significance, probably because of insufficient statistical power from small effective sample sizes (after removing missing observations) or high variability.

### Comparisons of complete remission and non-complete remission patients after treatments

In **Table 3**, we did not find statistically significant differences between the non-complete remission and complete remission groups for age, gender, tumor size, fasting plasma glucose, HbA1c, insulin, and C-peptide levels at baseline and after treatment. Yet, pre-treatment insulin levels appeared to be much higher in the non-complete remission group than in the complete remission group, with marginal statistical significance due to a large variability (212.57 ± 263.09 vs. 36.46 ± 34.03, *p* = 0.0624). Surgery achieved the highest complete remission rate for insulinoma while it was chosen as the first treatment modality (13/14, 92.86%, *p* = 0.0157), in patients who ever received it (14/15, 93.33%, *p* = 0.0157), and as one of the final treatment modalities (13/13, 100%, *p* = 0.0015).

Moreover, **Figure 2** (2) examines changes in glucose, HbA1c, insulin, and C-peptide levels before and after treatment between the non-complete remission (*n* = 4) and complete remission (*n* = 15) patients. Fasting plasma glucose levels increased significantly after treatment in both patient groups (*p* = 0.0024 and < 0.0001). HbA1c increased after treatment even in the non-complete remission group (*p* = 0.0388) and increased more in the complete remission group (*p* = 0.0003). Insulin levels did not decrease significantly after treatment in the non-complete remission group, although a notable reduction was observed, whereas they did in the complete remission group (*p* = 0.0193). Lastly, C-peptide levels decreased with borderline significance in both patient groups (*p* = 0.0789 and 0.0567).

### Predictors of insulinoma complete remission

**Figure 3:**
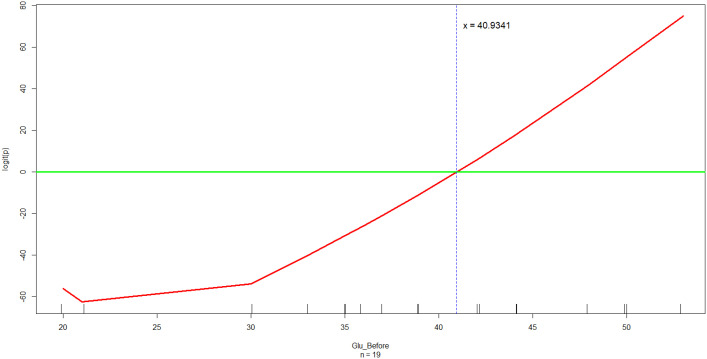
The multiple GAM plot for the effect of baseline glucose levels on the logit of insulinoma complete remission probability. This GAM plot was drawn based on the multiple GAM of insulinoma *complete remission* conditioning on the dummy variable of “ever surgery” to help us visualize the effect of baseline glucose levels in mg/dL (*X*-axis) on logit(*P*) (*Y*-axis), where logit(*P*) = log(*P*/(1−*P*)) and *P* was the probability of *complete remission* in insulinoma. The short vertical bars (called ‘rugs’) above the *X*-axis indicated where the observed *X* values were located. The red smoothed effect curve rose steadily with increases in baseline glucose levels (mg/dL), indicating an approximately linear relationship between baseline glucose levels (*X*-axis) and logit(*P*) (*Y*-axis) in the logistic regression model (**Table 4**). As explained in Section 2 of the **Supplementary Appendix**, the intersection of the horizontal green line and the red smoothed effect curve yielded an estimated optimal cut-off value of 40.9341 mg/dL for baseline glucose levels, at which the logit(*P*) would *not* increase or decrease. When the baseline glucose levels > 40.9341 mg/dL, the value of logit(*P*) increased, and then *P* increased accordingly, and *vice versa*. In other words, the *Y*-axis of this GAM plot indicated the rise or decline in the value of logit(*P*) (i.e., Δ logit(*P*)) with respect to the horizontal green line at Δ logit(*P*) = 0.

In **Table 4**, the Firth’s bias-reduced logistic regression analysis identified two statistically significant predictors of insulinoma complete remission: “Fasting plasma glucose (mg/dL) at baseline” (estimated adjusted odds ratio [aOR] = 1.2140, *p* = 0.0461) and “Ever surgery” (yes vs. no) (aOR = 296.0707, *p* = 0.0015). Surgery remained very effective for the complete remission of insulinoma. In addition, the lower the baseline fasting plasma glucose level, the lower the chance of complete remission. After adjusting for the effects of these two, other covariates — including treatment modality other than surgery, age, sex, tumor size, HbA1c, insulin, and C-peptide — were not significantly associated with complete remission in insulinoma. Moreover, the multiple generalized additive models (GAM) plot in **Figure 3** revealed that the smoothed effect curve of baseline fasting plasma glucose levels (mg/dL) on the logit of probability of complete remission in insulinoma rose steadily with increases in baseline fasting plasma glucose levels, indicating an approximately linear relationship between baseline fasting plasma glucose levels and logit(Probability(Complete Remission)) — especially for patients with baseline fasting plasma glucose levels > 40.9 mg/dL. The estimated area under the Receiver Operating Characteristic curve, *c* = 0.9667 > 0.7 (95% confidence interval [95% CI]: 0.8876−1.0), indicated an excellent fit of this Firth’s bias-reduced logistic regression model to the observed binary responses in the 19 insulinoma patients.

**Figure 4:**
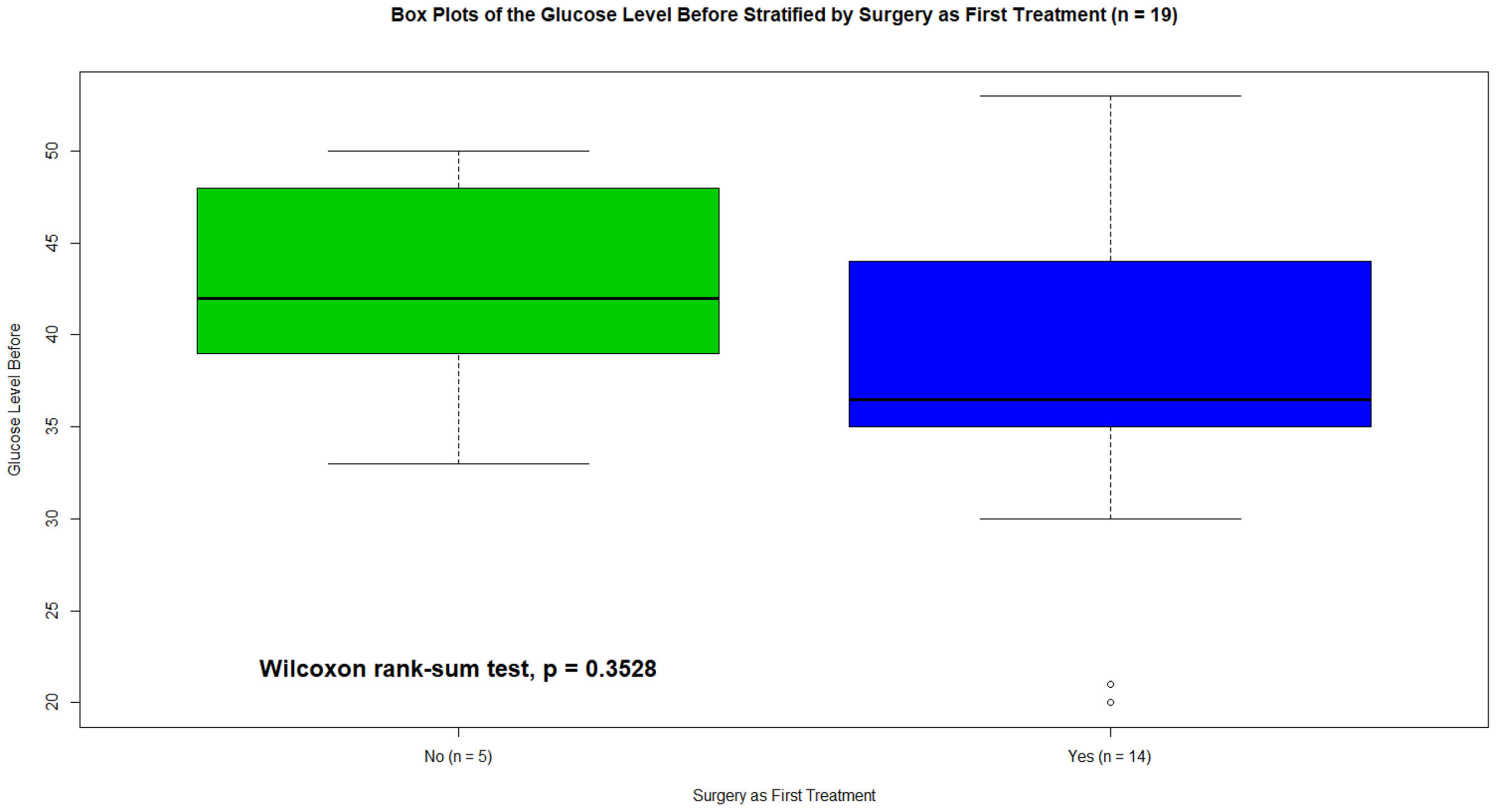
Box plots of baseline fasting plasma glucose (mg/dL) stratified by surgery as the first treatment strategy (*n* = 19). Comparison of the baseline fasting plasma glucose levels (mg/dL) between patients who did not receive surgery as first-line therapy and those who received surgery as first-line therapy showed different median values (42 mg/dL in the non-surgery group vs. 36 mg/dL in the surgery group), with overlapping interquartile ranges (IQRs). The two-tailed Wilcoxon rank-sum test comparing the medians of the two groups did not show a statistically significant difference (*p* = 0.3528). Among our patients with insulinoma, those with lower baseline fasting plasma glucose levels seemed to be more likely to choose surgery as initial treatment, but baseline fasting plasma glucose levels were also more variable in the initial surgical group.

As shown in **Figure 4**, baseline fasting plasma glucose levels were more variable in the initial surgery group, but those with lower baseline fasting plasma glucose levels appeared to be more likely to choose surgery as the initial treatment (median: 42 vs. 36 mg/dL), although the difference in baseline fasting plasma glucose levels was not statistically significant between these two patient groups (*p* = 0.3528). Surgery achieved the highest complete remission rate for insulinoma. Conditioning on “Ever surgery” (yes vs. no), lower baseline fasting plasma glucose levels led to a lower chance of complete remission (**Table 4**). Thus, as shown in **Table 3**, it seemed reasonable to see that the baseline fasting plasma glucose level did not differ significantly between the non-complete remission and complete remission patients (36.00 ± 11.75 vs. 39.27 ± 8.45, *p* = 0.6158).

## Discussion

### Treatment strategies

Although rare, insulinoma is the most common functional neuroendocrine tumor of the pancreas, typically presenting with symptomatic fasting hypoglycemia and, less commonly, post-prandial hypoglycemia. Surgical resection achieves a high complete remission rate and remains the standard of care in insulinoma. However, surgery carries notable post-operative risks, including pancreatic leakage, pancreatitis, hemorrhage, pseudocyst formation, intra-abdominal abscess, and new-onset DM (16, 17). Despite its effectiveness, surgery may not be feasible in all patients due to age, comorbidities, and personal preference, and in some cases, it cannot achieve complete remission or prevent recurrence (2, 18, 19).

Alternatively, EUS-guided ethanol injections have emerged as a minimally invasive rescue therapy, with a favorable safety profile, capable of inducing tumor necrosis and restoring euglycemia (19). It provides the advantage of quicker recovery and fewer complications, making it a valuable alternative or adjunct therapy in patients not suitable for repeat surgery or with residual disease following initial resection.

The technique of ethanol ablation has been successfully applied to treat renal cysts, hepatic cysts, hepatic solid tumors, and thyroid nodules (20–22). Its therapeutic mechanism involves coagulative necrosis induced by cellular dehydration, protein denaturation, and vascular occlusion (22). In 2007, a pilot study demonstrated that EUS-guided fine needle ethanol injection into the porcine pancreas could produce localized tissue necrosis without significant complications (23). Despite its promising therapeutic effect, ethanol ablation has several limitations, including potential for incomplete tumor destruction, recurrence requiring retreatment, and tumor progression during follow-up. Thus, a complete response should be confirmed by post-treatment contrast-enhanced imaging (24). Reported complications are generally mild, including transient abdominal pain, mild pancreatitis, and pancreatic hematoma (15). To minimize these risks, meticulous technique — using small ethanol volumes, careful intra-tumoral injection, and precise targeting — is crucial (25). Previous studies have used ethanol concentrations of 95−99% for tumors typically 1−2 cm in diameter (22, 25, 26). In 5 of our patients with insulinomas ranging in size from 0.9 to 1.5 cm, we successfully used 99.5% ethanol to treat them. They experienced immediate symptom relief and had significantly shorter post-procedural hospital stays. Yet, its long-term efficacy in insulinoma remains to be validated in larger prospective studies.

For patients who are poor surgical candidates or refuse to undergo surgery, medical therapy with diazoxide or SRLs such as octreotide and lanreotide is another therapeutic option. Although effective in some cases, medical therapy only provides partial control of symptoms and rarely leads to long-term remission (27).

A patient with multiple insulinomas in our cohort was treated with long-term octreotide LAR, a long-acting somatostatin analogue, and then achieved complete control of symptoms. This patient experienced a dramatic and sustained weight reduction. The weight decreased from 101 kg to 51 kg within 12 months of treatment initiation and remained stable over more than 100 months of follow-up. This case highlights the importance of individualized treatment strategies that consider tumor burden, patient comorbidities, and long-term treatment tolerance. Although SRLs are generally considered palliative, this case suggests that, in rare instances, they can offer long-lasting disease control and profound metabolic benefits (27).

### Diagnosis

Occult insulinomas present an ongoing diagnostic challenge. Cross-sectional imaging, such as CT or MRI, can miss small or sub-centimeter lesions, with reported false-negative rates exceeding 10%. Blind exploratory surgery is no longer recommended, as it may not identify the lesion and carries the risk of unnecessary complications. In these circumstances, SACST with hepatic venous sampling provides a highly accurate (up to 94%) preoperative localization strategy (28). A ≥ 19-fold increase in hepatic venous insulin levels relative to baseline is considered highly specific (99%) for insulinoma (29). In our 19 patients, SACST successfully localized the insulinoma in 4 out of 5 cases employed (80%), with failure only in a patient with multiple insulinomas.

EUS has also become an essential diagnostic tool for insulinoma. It offers nearly 100% sensitivity for detecting insulinomas < 1 cm when performed by experienced endosonographers (11, 14, 30). In our study, EUS was performed in all patients prior to ethanol ablation, serving as a key tool for both diagnosis and treatment guidance.

One of our cases faced the diagnostic challenge when drug-induced IAS coexisted with insulinoma, underscoring the importance of a thorough review of medications in the evaluation of unexplained hypoglycemia. This patient had a C-peptide-to-insulin molar ratio of less than 1, consistent with IAS. It occurred because autoantibody-bound insulin is not cleared efficiently, leading to disproportionately high insulin levels while C-peptide remained unaffected, and then resulting in a low molar ratio (31). Although rare, clopidogrel-induced IAS should be meticulously considered, particularly in East Asian populations, where specific HLA subtypes — such as HLA-DRB1*0406 — are known to confer increased genetic susceptibility to IAS (32).

Another patient presented multiple liver metastases at initial diagnosis, together with a large pancreatic tail tumor of 5.2 cm. He underwent an octreotide test, which resulted in severe paradoxical hypoglycemia immediately following injection. Nevertheless, he achieved reasonable glycemic control with everolimus and lanreotide, intermittently supplemented with rescue doses of diazoxide and two sessions of drug-eluting beads transarterial chemoembolization. He remained clinically stable for up to 59 months since diagnosis. His clinical course aligned with previous reports, suggesting that lanreotide may offer a more favorable glycemic profile than octreotide in some patients with insulinoma (5).

Among patients who took the octreotide tests or were treated directly with octreotide, 2 out of 8 cases (25.0%) experienced hypoglycemia in response, indicating variable efficacy or even paradoxical effects in some cases of insulinoma. While octreotide, an SRL, typically inhibits insulin secretion and is often used therapeutically for insulinomas, its effects can be unpredictable (33). In some patients, octreotide may preferentially suppress counter-regulatory hormones such as glucagon or growth hormone, thereby exacerbating hypoglycemia rather than alleviating it. This paradoxical response may also reflect tumor heterogeneity — specifically, variations in somatostatin receptor subtype expression, particularly SSTR2 and SSTR5, which influence octreotide’s binding and inhibitory effect on insulin secretion. Therefore, a positive response to the octreotide test should not be assumed to be universal, and careful monitoring during the test is warranted to prevent unintended adverse outcomes (5).

Finally, although not used in our cohort, emerging evidence from case series suggests that pasireotide, owing to its broader somatostatin receptor binding profile, may provide improved glycemic control in patients with insulinoma, particularly in those refractory to first-generation SRLs such as octreotide or lanreotide (34).

### Treatment outcomes

Fasting plasma glucose levels increased significantly in all three final treatment groups, with the smallest *p* value in the surgery group (*p* < 0.0001). These findings demonstrated the effectiveness of each treatment strategy in improving hypoglycemia. Surgery resulted in a significant increase in HbA1c (*p* = 0.0003) and a significant reduction in insulin levels (*p* = 0.0323), but ethanol ablation led to a significant decline in C-peptide (*p* = 0.0075). These results indicated that HbA1c and insulin level are more sensitive markers of the treatment effect than the C-peptide alone in insulinoma.

In our logistic regression analysis (**Table 4**), the baseline fasting plasma glucose level outperformed other biochemical markers (HbA1c, insulin, and C-peptide), tumor metrics (size and volume), and non-surgical treatment modalities in predicting curative outcomes in our insulinoma patients. Thus, baseline fasting plasma glucose level could serve as a useful biomarker for prognostication and treatment stratification in clinical practice. Although severe hypoglycemia reduced the chance of complete remission and was more common among surgery patients, surgical treatment still demonstrated an independent beneficial effect on complete remission in insulinoma after controlling for the effect of the baseline fasting plasma glucose level in the multivariable analysis.

Lower baseline fasting plasma glucose levels, especially < 40.9 mg/dL, signaled a more aggressive or treatment-resistant disease and led to greater resistance to insulinoma complete remission (**Figure 3**). In this sense, 40.9 mg/dL may be considered a threshold value for the baseline fasting plasma glucose level in the treatment of insulinoma. To our knowledge, this is the first study to report that pre-treatment fasting plasma glucose levels < 40.9 mg/dL are an important warning signal when planning to treat patients with insulinoma.

**Figure 5:**
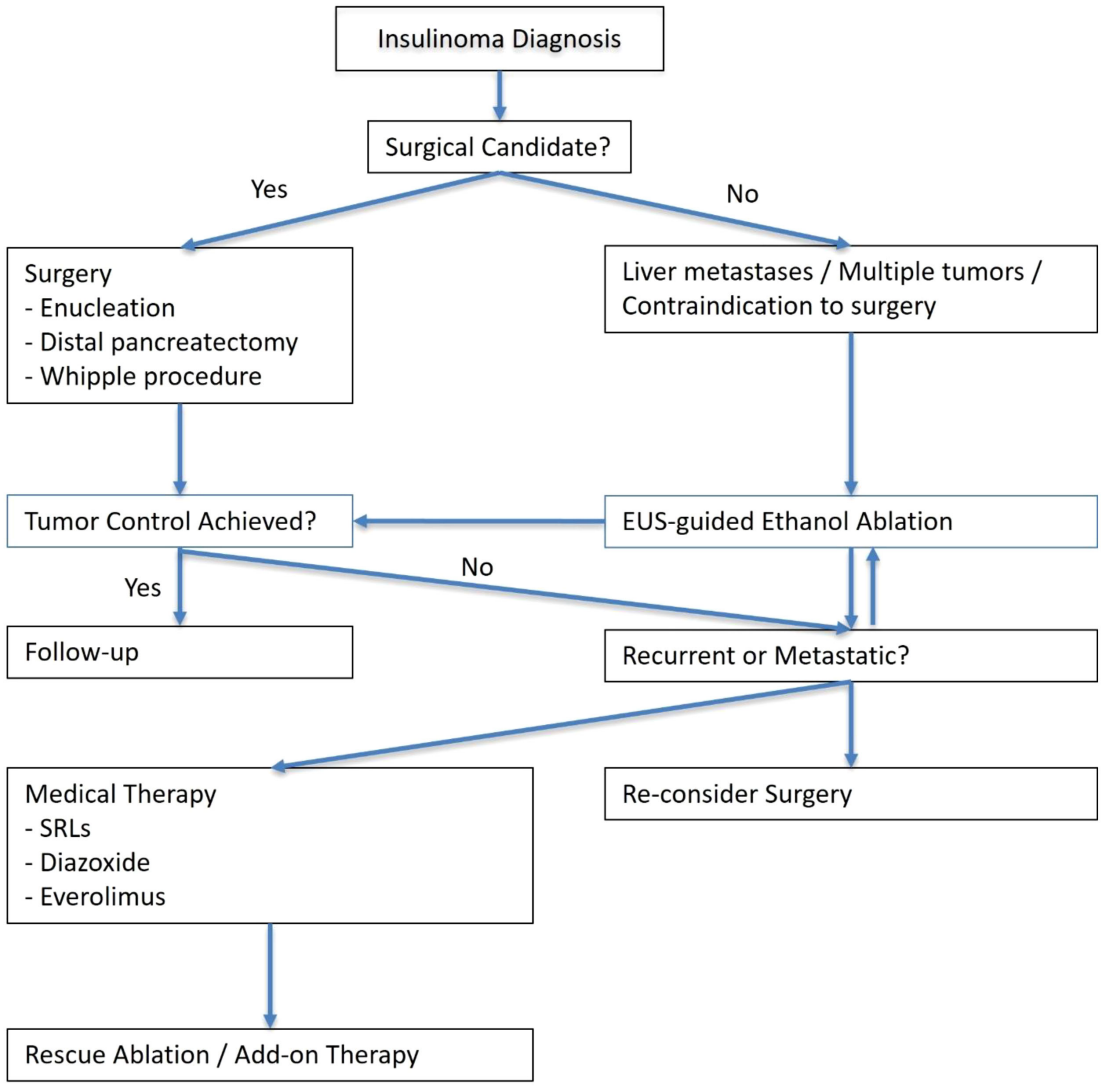
Management algorithm for insulinoma. This flowchart illustrates a stepwise clinical approach to the diagnosis and management of insulinoma. Following confirmation of insulinoma, surgical candidacy is assessed. Patients eligible for surgery proceed to definitive resection, including enucleation, distal pancreatectomy, or pancreaticoduodenectomy (Whipple procedure). Patients who are not surgical candidates due to liver metastases, multifocal disease, or other contraindications are considered for EUS-guided ethanol ablation. Tumor control is subsequently evaluated; patients achieving adequate control enter a follow-up phase, whereas those with persistent, recurrent, or metastatic disease undergo further stratification. Non-surgical candidates receive medical therapy, including somatostatin receptor ligands (SRLs), diazoxide, or everolimus, with consideration of rescue ablation or add-on therapy as needed. In selected cases with recurrent or metastatic disease who regain surgical eligibility, surgical intervention may be reconsidered. Directional arrows indicate clinical decision flow.

In **Figure 5**, we developed a clinical decision algorithm for the management of insulinomas based on the results of this study.

### Limitations

This retrospective study has three limitations. First, the overall sample size was small, particularly in the ethanol ablation and medical therapy groups, which limited statistical power and generalizability. Although some statistically significant findings were observed, these results should be interpreted with caution. Second, treatment decisions were influenced by patient-specific factors rather than standardized protocols. Given the retrospective chart reviews and non-randomized treatment allocation, baseline differences likely reflect selection bias in clinical decision-making. Hence, they were included in our logistic regression analysis, but comparisons between treatment groups should still be interpreted as associative rather than causal. Third, the study population was heterogeneous, with variability in comorbidities, tumor characteristics, and treatment regimens, further complicating direct comparisons of therapeutic effects. In particular, ethanol ablation was performed in selected patients who were not optimal surgical candidates or who required an alternative therapeutic approach. Therefore, treatment allocation was not randomized, and the observed differences among treatment modalities may have been influenced by patient selection and treatment heterogeneity, representing an important limitation of this retrospective study. Although the Firth’s bias-reduced logistic regression model demonstrated good apparent performance (*c* = 0.9667, 95% CI: 0.888–1.000), the predictive logistic regression model and warning sign of a baseline blood glucose level < 40.9 mg/dL should be considered exploratory and subject to verification in other patient cohorts. These three limitations underscore the need for large, prospective studies to validate the long-term efficacy and safety of surgery, EUS-guided ethanol ablation, and medical therapy in patients with insulinoma.

An additional limitation is that the definition of complete remission in this study may not completely exclude biochemical recurrence or late relapse. Although complete remission was defined as sustained biochemical euglycemia with resolution of hypoglycemic symptoms for at least 1 year after treatment without the need for anti-hypoglycemic medications or further therapeutic intervention, the retrospective design resulted in variability in the frequency and timing of biochemical assessments during follow-up.

## Conclusions

EUS-guided ethanol ablation represents a minimally invasive alternative to surgical resection, the current gold standard for treating insulinoma. It may provide rapid symptom relief with a favorable safety profile, particularly in patients who are not suitable surgical candidates. However, incomplete ablation or recurrence may necessitate subsequent surgical intervention, while ethanol ablation may also be considered following unsuccessful surgery.

The coexistence of insulin autoimmune syndrome (IAS) and variable responses to somatostatin receptor ligands highlights the need for individualized and multimodal treatment strategies. Lower baseline fasting plasma glucose levels (< 40.9 mg/dL) were associated with treatment resistance and may reflect more aggressive disease biology, potentially related to tumor burden.

Based on these findings, we propose a preliminary management algorithm for insulinoma. However, this algorithm and the identified glucose threshold should be considered exploratory and hypothesis-generating. Larger, prospective studies are warranted to validate these findings and to define the optimal role of each treatment modality in clinical practice.

## Data Availability

The raw data supporting the conclusions of this article will be made available by the authors, without undue reservation.
