# Supplementary material for: Clinical outcomes of medical therapy, EUS-guided ethanol ablation, and surgical resection in insulinoma: a clinical epidemiology study
Source: Front Endocrinol (Lausanne). 2026 Jul 27;17:1848169. doi: 10.3389/fendo.2026.1848169 (PMC13454529; doi:10.3389/fendo.2026.1848169)
Supplement: Supplementary file 1 [file DataSheet1.pdf]

Supplementary Appendix

1. Model-Fitting Techniques

The goal of regression analysis was to find one or a few parsimonious regression models that fit the observed data well for effect estimation and/or outcome prediction. To ensure a good quality of the analysis result, the model-fitting techniques for (1) *variable selection*, (2) *goodness-of-fit (GOF) assessment*, and (3) *regression diagnostics and remedies* were applied in our logistic regression analysis. Specifically, the modern stepwise variable selection procedure (with iterations between the *forward* and *backward* steps) was applied to obtain the best candidate final logistic regression model(s), using the 'My.stepwise' package of R.[1] All the univariate significant and non-significant relevant covariates (listed in Tables 2 and 3 of the text) and some of their interaction terms (i.e., *moderators*) were put on the variable list to be selected. The significance levels for *entry* (SLE) and *stay* (SLS) were set at 0.15 to be conservative. Then, with the aid of substantive knowledge, the best final logistic regression model was manually identified by removing the covariates with p value > 0.05 *one at a time* until all regression coefficients were significantly different from 0. Any discrepancy between the results of univariate analysis and multivariate analysis was likely due to the confounding effects of uncontrolled covariates in univariate analysis or the masking effects of intermediate variables (i.e., *mediators*) in multivariate analysis.

The GOF measures, (1) the estimated area under the receiver operating characteristic (ROC) curve (also called the c statistic) and (2) adjusted generalized R^2, and the Hosmer-Lemeshow GOF test were examined to assess the GOF of the fitted logistic regression model. The value of the c statistic ($0 \leq c \leq 1$) ≥ 0.7 suggests an acceptable level of discrimination power for logistic regression models. Yet, the values of adjusted generalized R^2 ($0 \leq R^2 \leq 1$), proposed by Nagelkerke,[2] are usually low — in our experience, adjusted generalized $R^2 \geq 0.30$ indicates an acceptable fit for the logistic regression model. The larger p-values of the Hosmer-Lemeshow GOF test imply better fits

of the logistic regression model, as they are unlikely to reject the null hypothesis of fitting well.

Simple and multiple *generalized additive models* (GAMs) were fitted to draw the GAM plots for detecting nonlinear effects of continuous covariates and then for identifying the appropriate cut-off point(s) to discretize continuous covariates, if necessary, during the stepwise variable selection procedure. Computationally, we used the vgam() function with the default values of the smoothing parameters (e.g., s(age, df=4, spar=0) for the cubic smoothing splines) in the 'VGAM' package to fit the GAMs for our binary responses, and then used the plotvgam() function of the same package to draw the smoothed-effect plots (called the GAM plots) for visualizing the linear or nonlinear effects of continuous covariates in R.[3,4,5]

If a separation (or high discrimination) problem occurred in logistic regression analysis, the logistf() function of the 'logistf' package would be used to fit Firth's bias-reduced logistic regression models with the profile penalized log-likelihood method in R.[6] Finally, the regression diagnostic tools for residual analysis, detection of influential cases, and check of multicollinearity were applied to discover any model or data problems. A variance-inflating factor (VIF) ≥ 10 for continuous covariates or VIF ≥ 2.5 for categorical covariates indicates the presence and source of multicollinearity among some covariates in the fitted logistic regression model. If any model or data errors were identified, we re-ran the regression analysis using the corrected model or data.

2. **The GAM Plots**

In our logistic regression analysis, simple and multiple *generalized additive models* (GAMs) were fitted to detect the nonlinear effects of continuous covariates and identify the appropriate cut-off point(s) for discretizing continuous covariates, if necessary, during the modern stepwise variable selection procedure. Computationally, the vgam() function (with the default smoothing parameter values) from the 'VGAM' package[3,4,5] was used to fit GAMs for binary responses in R. The GAM plot drawn according to a fitted simple or multiple GAM was named a simple or multiple GAM plot, respectively. According to the GAM fitted to a binary outcome variable, the curve drawn in the simple or multiple GAM plot helped us visualize the effect of a continuous covariate (X-axis) on logit(P) (Y-axis), where logit(P) = log(P/(1−P)) and P is the probability of insulinoma cure. The short vertical bars (called "rugs") above the X-axis indicated where the observed X values were located. In such a simple or multiple GAM plot, the intersection(s) between the horizontal green line and the red smoothed-effect curve yielded the estimated optimal cut-off value(s) of the X-axis, at which the value of logit(P) would *not* increase or decrease. When the covariate values > an estimated optimal cut-off value, the value of logit(P) increased or decreased, and then P increased or decreased accordingly, depending on how the red curve appeared, and *vice versa*. In other words, the Y-axis of this GAM plot actually indicated the rise or decline in the value of logit(P) (i.e., Δ logit(P)) with respect to the horizontal green line at Δ logit(P) = 0.

References

1. Hu, F. -C. My.stepwise: Stepwise variable selection procedures for regression analysis. R package, version 0.1.0, 2017 (https://CRAN.R-project.org/package=My.stepwise).
2. Nagelkerke N. A note on a general definition of the coefficient of determination. *Biometrika*, 1991; 78: 691−692.
3. Yee, T. W. and Wild, C. J. (1996). Vector generalized additive models. *Journal of the Royal Statistical Society*, Series B, 58(3): 481−493.
4. Yee, T. W. (2015). *Vector Generalized Linear and Additive Models: With an Implementation in R*. New York, NY: Springer Science+Business Media.
5. Yee, T. W. (2025). VGAM: Vector generalized linear and additive models. R package, version 1.1-13 (https://CRAN.R-project.org/package=VGAM).
6. Heinze, G., Ploner, M., Dunkler, D., Southworth, H., and Jiricka, L. (2025). logistf: Firth's bias-reduced logistic regression. R package, version 1.26.1 (https://CRAN.R-project.org/package=logistf).
